# Medical conduct and knowledge about physical activity counseling in the largest hospital complex in Latin America

**DOI:** 10.1016/j.clinsp.2025.100666

**Published:** 2025-05-07

**Authors:** Débora Borowiak Reiss, Ítalo Ribeiro Lemes, Hamilton Roschel, Bruno Gualano

**Affiliations:** a Applied Physiology and Nutrition Research Group – Center of Lifestyle Medicine, Faculdade de Medicina, Universidade de São Paulo (FMUSP), São Paulo, SP, Brazil; b Instituto de Ortopedia do Hospital das Clínicas da Faculdade de Medicina da Universidade de São Paulo, São Paulo, SP, Brazil; c Fleury Group, Brazil; d Department of Physical Therapy, Faculdade Israelita de Ciências da Saúde Albert Einstein (FICSAE), São Paulo, SP, Brazil; e Department of Physical Therapy, São Paulo State University (UNESP), Presidente Prudente, SP, Brazil

**Keywords:** Physical activity, Medical education, Counseling

## Abstract

**Objective:**

To characterize practices and knowledge regarding PA counseling at a large tertiary hospital.

**Design:**

Cross-sectional observational study.

**Participants and setting:**

Physicians with an active medical practice within the university hospital were invited to respond to an online survey.

**Interventions:**

A questionnaire designed to capture information on medical training, life habits and knowledge about PA.

**Main outcomes measures:**

Sedentary behavior, clinical practice and self-efficacy were assessed by Likert-type questions, whereas regular practice, previous training, and knowledge of PA were evaluated through dichotomous questions (answers: “yes or no” or “true or false”).

**Results:**

Response rate was 11.5 % (50.6 % female, 44.9 ± 12.8 years), 67.7 % were physically active, and 63.3 % had 4 to 8 h/day of sedentary behavior. Only 40.7 % had previous training on PA, 99.7 % recognized the importance of recommending PA, but only a small part (48.4 %) recommended PA in all consultations (the main barrier was lack of specific training). In addition, 69.2 %, 58.7 % and 65.5 % rated their abilities to collect PA history, assess contraindications, and engage the patient as good/excellent, respectively. Knowledge of PA was rated as low to very low, with correct answers ranging from 49 % to 17 % and as low as ∼10 % (when considering the pediatric population).

**Conclusion:**

Although physicians recognize the importance of PA counseling and have high perceived self-efficacy in relation to counseling skills, few put them into practice. This may be due to insufficient training on PA and/or the complexity of services provided at the tertiary hospital. The present results corroborate the need for improvements in medical training on PA.

## Highlights

• PA training is allied to assessing, knowledge, and recognizing skills in PA counseling.
• Training on PA is the key for physicians to be confident in their counseling abilities.
• High self-efficacy for PA counseling is associated with willingness to prescribe PA.
• Major gaps in physicians’ knowledge of PA recommendations were identified.

## Introduction

The benefits of regular physical activity (PA) in preventing chronic diseases, reducing premature mortality, promoting overall health, and improving quality of life are well established.^1^^,^^2^ Clinical guidelines from various medical fields consistently recommend regular PA as the primary treatment for a wide range of diseases.^3^ Notwithstanding, the prevalence of physical inactivity in the general population has been steadily rising over the past decade, accompanied by increasing incidence of chronic diseases and healthcare costs.^4^

One of the roles of physicians is to educate patients about general health, including promoting PA.^3^^,^^5^ However, several barriers have been identified that hinder PA counseling and prescription by most professionals,^6^ with lack of knowledge at the top of the list.^7^ This may be related to the lack of specific training in this field in most medical school programs. Furthermore, this subject is almost non-existent during medical residency.^8^

PA counseling has been defined as an efficient^9^ and cost-effective^10^ strategy to improve one’s overall health and well-being. Incorporating high-quality PA counseling and prescriptions into the national health system could lead to substantial savings and a more equitable distribution of health resources, ultimately improving health and quality of life of the population. Therefore, to lay the foundation for evidence-based public policies centered on the promotion of PA, it is crucial to evaluate physicians’ knowledge and practices on this matter.

The aim of this study was to assess physicians’ knowledge and willingness to prescribe PA at a large tertiary hospital. The authors also identified facilitators and barriers to the recommendation of PA and evaluated the associations between perceived self-efficacy and performance in PA knowledge tests.

## Methods

This is a cross-sectional survey. The study was approved by the local ethics committee (CAAE 20231419.2.0000.0068). The manuscript was described following the STROBE Statement.

### Setting and participants

The Clinical Hospital of the School of Medicine of the University of São Paulo (HCFMUSP) is a large tertiary hospital located in São Paulo, Brazil. It offers 2400 beds, distributed among eight specialized institutes and two assisting hospitals. Physicians with active registration at the HCFMUSP complex (*n* = 5479) were invited to respond to an online questionnaire. The authors included physicians involved in assistance and teaching roles in medical residency and graduation programs while excluding those who work solely in administrative roles or in specialties that do not involve patients able to engage in PA (e.g., Intensive Care, Emergency Medicine, Laboratory Medicine, Clinical Pathology, Palliative Medicine, Digestive Endoscopy, etc.). Physicians who work solely on surgical and therapeutic procedures and interventions (e.g., Anesthesiology, Digestive Endoscopy, Gynecological Endoscopy, Radiotherapy, etc.), were also excluded. Prior to their participation, all respondents provided informed consent.

The Research Electronic Data Capture (REDCap) system, a secure web-based instrument for data collection and management, was used for this study. The questionnaire included questions related to medical training, lifestyle habits, and knowledge about PA. It underwent a thorough evaluation by specialists to ensure proper content, consistency, and coherence. To enhance the response rate, the questionnaire was sent by e-mail at least five times to each physician over three different periods.

### Survey

The survey comprised a combination of open-ended, multiple-choice, dichotomous (‘yes or no’) and Likert-type questions (e.g., ‘always; most times; a few times; never’), and was divided into the following sections:

- Regular practice (assessed through dichotomous “yes or no” questions) and sedentary behaviour (assessed by Lykert-type questions – i.e., up to 4 hours’; “>4 to 8hours”; “>8 to 12 h”; “>12 to 16 h”; “>16 h”).
- Clinical practice (the frequency of evaluation and recommendation of PA was assessed by Likert-type questions ‒ i.e., ‘always; most times; a few times; never’ and dichotomized into never/few times vs. most times/always), and previous training on PA (assessed through dichotomous “yes or no” questions).
- Knowledge about PA recommendations for the general population was assessed from an open question about the minimum weekly minutes of moderate and vigorous PA and the weekly frequency of strength training. The variables were dichotomized into correct vs. incorrect, and categorized in the regression analyses as 0 vs. 1, 2 or 3 correct answers. In addition, knowledge about PA for chronic diseases was assessed by false vs. true questions and then dichotomized into < 80 % vs. ≥ 80 % correct answers in the regression analyses.
- Perceived self-efficacy was assessed based on questions about how physicians perceive their abilities to collect PA history, assess contraindications to PA practice, prescribe PA, and convince the patient to practice PA, which was assessed by Likert-type questions (i.e., ‘excellent; good; regular; poor’), being dichotomized into poor/regular vs. good/excellent.

The response time for completing the questionnaire was approximately ten minutes (Supplementary Material).

### Statistical analysis

Continuous variables are presented as mean ± standard deviation, assuming data normality. Categorical variables are presented as absolute and relative ( %) values. Comparisons between sections were evaluated using the Chi-Square Test. The association between sex, age, time since graduation, being physically active, sedentary time, previous training on PA, specialty and residency area and clinical practice, knowledge, and self-efficacy was tested using adjusted binary logistic regression models and presented with Odds Ratio (OR) values and 95 % Confidence Intervals (95 % CI). Statistical significance was set at *p* < 0.05.

## Results

**Fig. 1:**
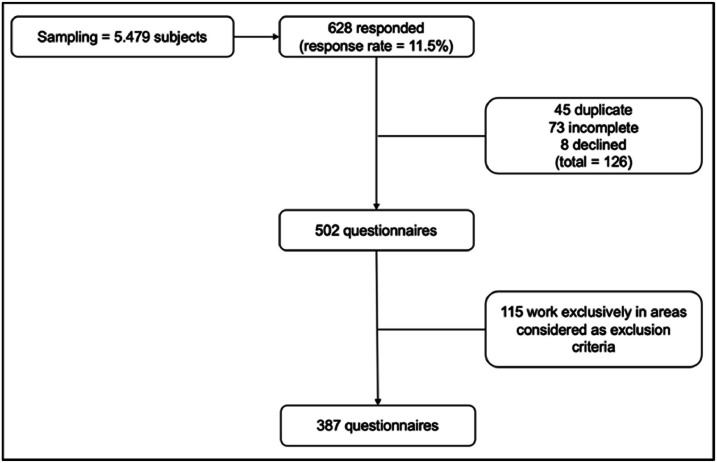
Flowchart of the total of evaluated responses.

The response rate was 11.5 % (628/5479), and a total of 387 valid questionnaires were analyzed (Fig. 1). Participants’ age was 44.9 ± 12.8 years on average, and 50.6 % were women. Among respondents, 67.7 % reported engaging in regular PA, while 63.3 % reported sitting for 4 to 8 h per day. Regarding academic background, 26.4 % of the participants completed their graduation between 2010 and 2019, and the vast majority (83.4 %) graduated from universities located in the state of São Paulo. Only 15.0 % were in residency programs at the time of data collection, and 355 (86.8 %) were specialist physicians, of whom 243 (72.5 %) worked in clinical areas, while the remainder worked as surgeons (Table 1).

**Table 1:** Demographic characteristics (*n* = 387) and academic background (*n* = 386).

	n	( %)
Sex		
Masculine	191	(49.4)
Feminine	196	(50.6)
Age groups		
21 to 30 years	64	(16.5)
31 to 40 years	89	(23.0)
41 to 50 years	106	(27.4)
51 to 60 years	65	(16.8)
61 to 70 years	58	(15.0)
71 to 80 years	5	(1.3)
Regular PA practice		
No	125	(32.3)
Yes	262	(67.7)
Change (PA practice) after the beginning of the pandemic		
Changed to more	81	(20.9)
Changed to less	176	(45.5)
Did not change	130	(33.6)
Time spent sitting (hours/day)		
Up to 4 h	38	(9.8)
From 4 to 8 h	245	(63.3)
From 8 to 12 h	88	(22.7)
From 12 to 16 h	12	(3.1)
>16 h	4	(1.0)
Change (sedentary time) after the beginning of the pandemic		
Changed to more	172	(44.4)
Changed to less	17	(4.4)
Did not change	198	(51.2)
Year of graduation		
1970 to 1979	30	(7.8)
1980 to 1989	61	(15.8)
1990 to 1999	81	(21.0)
2000 to 2009	97	(25.1)
2010 to 2019	102	(26.4)
2020 to 2021	15	(3.9)
Region where graduated		
North	9	(2.3)
North East	35	(9.1)
Midwest	9	(2.3)
Southeast	322	(83.4)
South	11	(2.9)
Residents		
No	328	(85.0)
Yes	58	(15.0)
Have any specialty		
No	51	(13.2)
Yes	335	(86.8)

PA, Physical Activity.

### Clinical practice and training on PA counseling

Among the participants, 40.7 % reported having received some training in PA counseling during their medical education. Whilst 99.7 % recognized the importance of recommending PA, only 31.9 % stated assessing PA levels in all opportunities, and 48.4 % reported always recommending PA (Table 2). Interestingly, even though 51.6 % did not consistently recommend PA, only 6.8 % provided responses about the barriers related to PA counseling (Table 2).

**Table 2:** Clinical practice.

	n	( %)
Profile of patients (*n* = 386)		
Only pediatric	26	(6.8)
Adults and/or elderly	268	(69.4)
All age groups	92	(23.8)
Evaluates PA level and sedentary time (*n* = 382)		
Always	122	(31.9)
Most of the time	168	(44.0)
Rarely	79	(20.7)
Never	13	(3.4)
Considers it importante to recommend PA (*n* = 382)		
No, because it does not interfere with the treatment	1	(0.3)
Yes, AP is na adjunct to treatment	131	(34.3)
Yes, PA is first-line treatment	250	(65.4)
Recommends PA (*n* = 382)		
Always	185	(48.4)
Most of the time	171	(44.8)
Rarely	25	(6.5)
Never	1	(0.3)
Barriers to recommending PA (*n* = 51)		
Lack of time	12	(23.5)
Lack of specific training	16	(31.4)
Not part of medical job	0	(0.0)
Not an important clinical parameter	1	(2.0)
PA can make some symptoms worse	2	(3.9)
Patients do not adhere to recommendations	7	(13.7)
My patients have contraindications to the practice of PA	2	(3.9)
Patients have nowhere to practice PA	7	(13.7)
Others	4	(7.8)

PA, Physical Activity.

### Self-efficacy, knowledge about PA and sedentary behavior, and the need for medical training

**Fig. 2:**
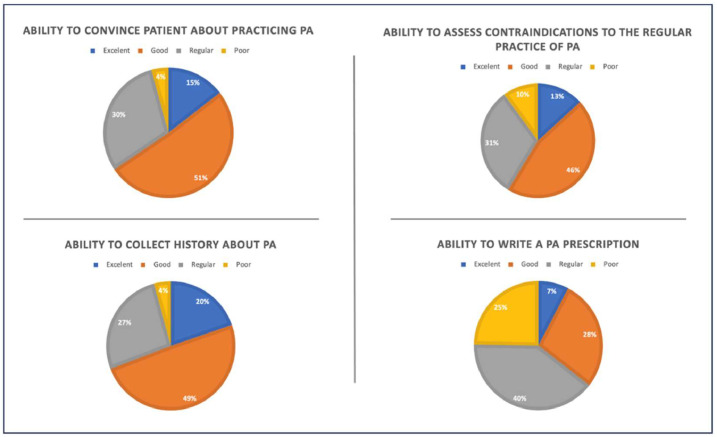
Assessment of perceived self-efficacy regarding the recommendation and prescription of PA (*n* = 380).

Regarding self-efficacy, 64.4 % of respondents reported regular or poor ability in prescribing PA (Fig. 2). In relation to PA knowledge, the rates of correct answers for moderate PA, vigorous PA, and strength training were 49 %, 17.3 % and 36 %, respectively (Table 3). Most participants (64.2 %) could not define PA properly, confusing it with exercise, while only 47 % answered most questions about PA correctly (Table 3). The vast majority of the participants (87.3 %) believed PA should be a mandatory subject in medical training and 11.9 % thought it should be optional.

**Table 3:** Knowledge about PA and sedentary behavior.

	Expected answer	Correct answers	( %)
PA recommendations (*n* = 375)			
At a minimum, how many minutes of moderate PA should be practiced over the course of a week?	150 min/sem^1^^,^^2^	187	(49.9)
At a minimum, how many minutes of vigorous PA should be practiced over the course of a week?	75 min/sem^1^^,^^2^	65	(17.3)
Strength exercises should be practiced at least how many days a week?	days/sem^1^^,^^2^	135	(36.0)
For children and adolescents, at a minimum, how many minutes of moderate to vigorous PA should be practiced per day?	60 min^1^^,^^2^	39	(10.5)
Recommendations on sedentary time for the pediatric population (*n* = 372)			
For children aged 6 to 10, sedentary time during leisure time (TV, computer, Cell phone, etc.) should be limited to how many minutes a day?	120 min^3^	71	(19.1)
For adolescents aged 11 to 18, sedentary time during leisure time (TV. computer. Cell phone. etc.) should be limited to how many minutes a day?	180 min^3^	37	(9.9)
General knowledge about PA (*n* = 374)			
PA should not be recommended for patients with chronic pain	False	369	(98.7)
Strength exercises should not be recommended for patients younger than 12-years old	False	239	(63.9)
A person who does not reach the PA recommendations is considered sedentary	False	209	(55.9)
PA should not be recommended for patients who present with fever, leuokepnia, anemia or malnutrition	True	150	(40.1)
PA should be recommended for patients with chronic diseases, but sports practice should not	False	351	(93.9)
Very low-intensity PA, such as light walking. promotes health benefits	True	286	(76.5)
PA should not be recommended for patients with hypertension, type 2 diabetes, and heart failure	False	368	(98.4)
Exercise is any movement produced by muscle contraction that results in energy expenditure above baseline levels	False	134	(35.8)
Patients with chronic diseases should always avoid sedentary behaviour, unless clinical descompensation	True	320	(85.6)
Exacerbation of inflammation and muscle damage are adverse effects of PA in patients with chronic diseases	False	342	(91.4)

Reference: WHO 2020[1]; MS 2021[2]; SBP 2019[3].

PA, Physical Activity; TV, Television; min/sem, minutes per week; days/sem, days per week; min, minutes.

### Factors associated with medical knowledge about PA and perceived self-efficacy

Table 4 presents the factors associated with physicians’ willingness to assess and prescribe PA. Previous training on PA (OR = 1.94 [1.15 to 3.26]) and clinical specialty (OR = 2.25 [1.29 to 3.91]) were significantly associated with routine assessment of PA, independent of confounding variables. Furthermore, completing a residency program (OR = 4.33 [1.74 to 10.76]) was associated with correctly answering questions about moderate PA. Previous training on PA (OR = 2.73 [1.52 to 4.90]) and completing a residency program (OR = 3.63 [1.46 to 9.04]) were associated with correctly answering questions about vigorous PA.

**Table 4:** Association between studied factors and PA assessment or counseling.

	*Assesses PA most of the time or always*	*Recommend PA most of the time or always*
Sex		
Male	Reference	Reference
Female	0.94 [0.56‒1.58]	1.36 [0.57‒3.24]
Age	1.05 [0.94‒1.18]	0.99 [0.86‒1.14]
Graduation time (years)	0.97 [0.87‒1.09]	1.06 [0.92‒1.22]
Practice PA regularly		
No	Reference	Reference
Yes	1.27 [0.76‒2.13]	1.46 [0.63‒3.37]
*Sedentary time (hours)*	1.11 [0.77‒1.59]	0.83 [0.47‒1.47]
Previous training		
No	Reference	Reference
Yes	1.94 [1.15‒3.26]^a^	1.27 [0.54‒2.98]
Specialty		
Surgical	Reference	Reference
Clinical	2.25 [1.29‒3.91]^b^	1.84 [0.75‒4.51]
Residency		
No	Reference	Reference
Yes	0.85 [0.38‒1.93]	2.41 [0.62‒9.34]

PA, Physical Activity; Model adjusted by sex, age, training time, regular PA practice, sedentary time, previous training, specialty and residency.

a < 0.05.

b < 0.01.

Table 5 presents the factors associated with physicians’ knowledge. Medical residency (OR = 4.33 [1.74 to 10.76]) was associated with answering correctly about moderate PA. Previous training on PA (OR = 2.73 [1.52 to 4.90]) and residency (OR = 3.63 [1.46 to 9.04]) were associated with answering correctly about vigorous PA.

**Table 5:** Association between studied factors and PA knowledge.

	PA for general population			PA and sedentary time in pediatric population	PA and chronic diseases
	Minimum moderate PA per week	Minimum vigorou PA per week	Minimum strength PA per week		
	150 min	75 min	2 days	Get at least 1 out of 3 questions correct	Grade ≥80 %
Sex					
Male	Reference	Reference	Reference	Reference	Reference
Female	1.26 [0.79‒2.01]	1.09 [0.60‒1.99]	1.42 [0.90‒2.24]	1.48 [0.91‒2.41]	0.90 [0.58‒1.39]
Age	0.93 [0.87‒1.01]	0.96 [0.87‒1.05]	0.96 [0.87‒1.05]	0.93 [0.83‒1.05]	0.94 [0.86‒1.03]
Graduation time (years)	1.04 [0.96‒1.12]	1.04 [0.94‒1.14]	1.05 [0.95‒1.16]	1.08 [0.96‒1.21]	1.06 [0.97‒1.16]
Practice PA regularly					
No	Reference	Reference	Reference	Reference	Reference
Yes	1.26 [0.78‒2.01]	2.36 [1.19‒4.69]	1.60 [0.99‒2.56]	1.07 [0.65‒1.75]	0.87 [0.56‒1.36]
*Sedentary time (hours)*	1.04 [0.76‒1.42]	0.84 [0.55‒1.30]	0.96 [0.71‒1.31]	0.86 [0.62‒1.21]	1.05 [0.78‒1.41]
Previous training					
No	Reference	Reference	Reference	Reference	Reference
Yes	1.18 [0.75‒1.86]	2.73 [1.52‒4.90]^b^	1.17 [0.75‒1.82]	1.17 [0.73‒1.87]	1.19 [0.78‒1.81]
Specialty					
Surgical	Reference	Reference	Reference	Reference	Reference
Clinical	1.54 [0.91‒2.62]	1.34 [0.64‒2.81]	1.05 [0.62‒1.78]	1.04 [0.60‒1.83]	1.33 [0.81‒2.20]
Residency					
No	Reference	Reference	Reference	Reference	Reference
Yes	4.33 [1.74‒10.76]^b^	3.63 [1.46‒9.04]^b^	0.92 [0.42‒2.00]	1.29 [0.58‒2.91]	1.37 [0.67‒2.84]

PA, Physical Activity; Model adjusted by sex, age, training time, regular PA practice, sedentary time, previous training, specialty and residency.

* < 0.05.

b < 0.01.

An exploratory, *post-hoc* analysis revealed significant associations between being physically active and good/excellent ability to collect PA history (OR = 1.93 [1.20 to 3.09]), prescribe PA (OR = 1.88 [1.14 to 3.10]), and convince the patient to engage in PA (OR = 1.72 [1.09 to 2.73]). In addition, prior training on PA showed significant associations with the following abilities: collecting PA history (OR = 2.74 [1.66 to 4.50]), assessing contraindications to PA practice (OR = 4.40 [2.71 to 7.13]), prescribing PA (OR = 4.19 [2.63 to 6.68]), and convincing the patient to perform PA (2.04 [1.28 to 3.25]) (Table 6).

**Table 6:** Association between studied factors and perceived self-efficacy skills.

	Collect history about PA	Evaluate contraindications to PA practice	Write a PA prescription	Convince the patient to practice PA
	Good/Excellent	Good/Excellent	Good/Excellent	Good/Excellent
Sex				
Male	Reference	Reference	Reference	Reference
Female	0.91 [0.56‒1.48]	0.96 [0.60‒1.52]	0.99 [0.61‒1.60]	0.72 [0.45‒1.16]
Age	1.04 [0.94‒1.15]	1.00 [0.92‒1.08]	1.02 [0.95‒1.10]	1.03 [0.94‒1.13]
Graduation time (years)	0.96 [0.87‒1.07]	1.04 [0.96‒1.13]	1.00 [0.93‒1.08]	0.99 [0.91‒1.08]
Practice PA regularly				
No	Reference	Reference	Reference	Reference
Yes	1.93 [1.20‒3.09]^b^	1.08 [0.67‒1.73]	1.88 [1.14‒3.10]a	1.72 [1.09‒2.73]^a^
*Sedentary time (hours)*	1.07 [0.76‒1.49]	0.91 [0.66‒1.25]	0.95 [0.69‒1.32]	0.96 [0.69‒1.32]
Previous training				
No	Reference	Reference	Reference	Reference
Yes	2.74 [1.66‒4.50]^b^	4.40 [2.71‒7.13]^b^	4.19 [2.63‒6.68]^b^	2.04 [1.28‒3.25]^b^
Specialty				
Surgical	Reference	Reference	Reference	Reference
Clinical	1.26 [0.73‒2.18]	1.25 [0.73‒2.13]	1.04 [0.60‒1.82]	1.37 [0.81‒2.34]
Residency				
No	Reference	Reference	Reference	Reference
Yes	0.75 [0.34‒1.68]	1.42 [0.65‒3.08]	1.30 [0.57‒2.92]	0.83 [0.39‒1.78]

PA, Physical Activity; Model adjusted by sex, age, training time, regular PA practice, sedentary time, previous training, specialty and residency.

a < 0.05.

b < 0.01.

### Perceived self-efficacy and performance on knowledge tests

There was a significant association between one’s perceived skills in collecting PA history as good/excellent (*p* = 0.048) and prescribing PA (*p* = 0.021) with correctly answering questions about moderate PA (Table 7). In addition, perceived good/excellent skills in taking PA history (*p* = 0.001), assessing contraindications to PA practice (*p* = 0.003), and prescribing PA (*p* = 0.001) were associated with correctly answering questions about vigorous PA. Finally, perceived good/excellent abilities to assess contraindications to PA practice (*p* = 0.001) and prescribing PA (*p* = 0.014) were associated with correctly answering about strength training.

**Table 7:** Association between perceived self-efficacy with knowledge about PA recommendations for the general population.

Self-efficacy skills	Knowledgement											
	Moderate PA		Vigorous PA		Strenght PA		PA general population		PA and sedentary time for pediatric population		PA for chronic disesas	
	Correct		Correct		Correct		Get at least 1 out of 3 questions correct				Grade ≥80 %	
	n ( %)	p	n ( %)	p	n ( %)	p	n ( %)	p	n ( %)	p	n ( %)	p
*Collect history about PA*												
Poor or regular	49 (26.2)	0.048	9 (13.8)	0.001	38 (28.1)	0.381	71 (28.0)	0.070	29 (27.6)	0.388	51 (29.0)	0.422
Good or excellent	138 (73.8)		56 (86.2)		97 (71.9)		183 (72.0)		76 (72.4)		125 (71.0)	
*Evaluate contraindications to PA practice*												
Poor or regular	71 (38.0)	0.187	16 (24.6)	0.003	41 (30.4)	0.001	97 (38.2)	0.073	42 (40.0)	0.731	64 (36.4)	0.060
Good or excellent	116 (62.0)		49 (75.4)		94 (69.6)		157 (61.8)		63 (105)		112 (63.6)	
*Write a PA prescription*												
Poor or regular	111 (59.4)	0.021	31 (47.7)	0.001	77 (57.0)	0.014	157 (61.8)	0.055	58 (55.2)	0.013	109 (61.9)	0.245
Good or excellent	76 (40.6)		34 (52.3)		58 (43.0)		97 (38.2)		47 (44.8)		67 (38.1)	
*Convince the patient to practice PA*												
Poor or regular	67 (35.8)	0.637	16 (24.6)	0.061	41 (30.4)	0.190	88 (34.6)	0.990	30 (28.6)	0.156	60 (34.1)	0.878
Good or excellent	120 (64.2)		49 (75.4)		94 (69.6)		166 (65.4)		75 (71.4)		116 (65.9)	

PA, Physical Activity.

In an exploratory analysis to assess possible associations between perceived self-efficacy and knowledge about PA recommendations for specific populations, the authors observed an association between perceiving the ability to prescribePA as good/excellent (*p* = 0.013) with correctly answering at least one question about PA and sedentary time in the pediatric population (Table 7).

## Discussion

### Previous training in PA counseling and clinical practice

During medical education, only 40.7 % of physicians reported having received some training in PA counseling. The present findings are consistent with early observations,^11^ suggesting that there has been limited progress regarding the inclusion of specific training on PA counseling in medical education over the years. In 1992, only 3 % of physicians had taken a higher-level course related to the subject,^12^ and by 2014, this number had only increased to 11 %.^13^ More recently, in Brazil, a study found that only 15.2 % of physicians had previously participated in a course on PA^14^ and 17.6 % of physicians had previous training on PA counseling.^15^

The majority of physicians in this study (99.7 %) consider it important to recommend PA, but only 31.9 % assess the level of PA of their patients, and 48.4 % recommend PA in all appointments. These findings are consistent with those from the 1980s and 1990s.^12^^,^^16^^,^^17^

While few participants answered about the barriers to PA counseling, the main ones identified were a lack of time and specific training, which align with the predominant barriers cited in the literature.^18^, ^19^, ^20^, ^21^, ^22^, ^23^, ^24^ Other barriers mentioned include low remuneration,^18^, ^19^, ^20^ lack of patient interest in PA,^18^, ^19^, ^20^, ^21^^,^^23^, ^24^, ^25^ patient preference for drug treatment,^18^^,^^21^, ^22^, ^23^^,^^25^ prioritization of other preventive measures,^19^^,^^21^, ^22^, ^23^ not feeling qualified to discuss exercise,^22^^,^^24^ and patient’s safety concerns.^24^

As previous training on PA associates with actual PA recommendation, knowledge about guidelines, with perceived self-efficacy in PA counseling, incorporating comprehensive training on PA into medical education curricula is of utmost importance.

### Self-efficacy and knowledge about PA and sedentary behavior

The high self-efficacy regarding skills related to PA counseling in this study is consistent with previous findings.^13^^,^^14^^,^^26^^,^^27^ However, regarding prescribing PA, 64.4 % reported fair/poor ability, which is similar to other investigations.^28^, ^29^, ^30^ This finding is concerning, given the evidence suggesting that written prescriptions are more effective in increasing PA levels compared to simple verbal advice.^31^

Medical knowledge about PA appears to be inadequate, as most physicians were unaware of PA recommendations, corroborating previous studies.^26^^,^^28^^,^^32^ The lack of basic knowledge on the subject is evident, with 64.2 % being unable to correctly define the concept of PA. In fact, Yonis et al.^25^ reported that only 16.1 % of physicians considered to have good knowledge about PA recommendations. Indeed, while 99.7 % of physicians recognized the importance of recommending PA, 34.3 % still considered it as an adjuvant treatment, despite well-known evidence indicating that PA is the first-choice treatment for numerous diseases.^33^, ^34^, ^35^

Addressing this issue is another reason to train healthcare professionals in PA counseling, incorporating the topic into medical education curricula, and actively encouraging students and physicians to adopt active lifestyles. By implementing these measures, the authors can increase awareness and ensure the successful implementation of PA guidelines.^32^^,^^36^^,^^37^

### Factors related to clinical practice, medical knowledge, and perceived self-efficacy on PA counseling

The authors found that previous training on PA and clinical specialty were positively associated with the assessment of patients’ PA levels. A similar result was reported by Silva et al.,^24^ who observed that physicians with prior PA training were more likely to encourage their patients to engage in PA (31.3 % vs. 24.3 %, *p* < 0.05) and longer duration exercise sessions (22.9 % vs. 13.8 %, *p* < 0.005). This suggests that previous PA training influences physicians to be more willing to advise patients on PA.^24^

In Brazil, Moraes et al.^38^ showed that professionals more likely to advise on PA were those with academic experience in primary health care (OR = 2.68; 95 % CI: 1.32 to 5.92) and those who were more physically active (OR = 1.80; 95 % CI: 1.01 to 3.27).

The authors were unable to identify an association between gender and being physically active in counseling patients on PA. However, Hall et al.^39^ reviewed the literature and found five studies showing that female physicians were more likely to evaluate PA compared to male physicians, and four studies indicating that physically active professionals were more likely to advise PA to their patients.

Furthermore, the present study revealed that engaging in PA and previous training on the subject were related to most self-efficacy skills. Similar findings have been previously reported. For instance, Souza-Neto et al.^14^ showed that physicians with higher levels of self-efficacy (OR = 1.79; 95 % CI: 1.18 to 2.68) and a positive attitude (OR = 1.69; 95 % CI: 1.11 to 2.45) toward counseling PA were more likely to advise on PA.

In a recent literature review, Hall et al.^39^ found four studies supporting the notion that physicians with a positive assessment of their capabilities and/or self-efficacy were more likely to advise PA to their patients. This reinforces the idea that physically active physicians are more likely to advise more frequently and with more confidence about the importance of PA to their patients.^37^ Thus, knowing that ‘being physically’ active and mainly ‘having had previous training on PA’ are directly related to clinical practice, knowledge, and self-efficacy, it is plausible to suggest that increasing medical knowledge on PA recommendations and self-efficacy in counseling skills may help physicians promoting PA. To our knowledge, this study was a pioneer in exploring the relationship between perceived self-efficacy and medical knowledge about PA. Of relevance, the authors found that physicians who perceived a high level of self-efficacy tended to respond correctly to questions about PA recommendations. Moreover, those who reported good/excellent ability in prescribing PA were more likely to provide accurate responses regarding PA.

In another study^40^ where familiarity with national PA guidelines was assessed, only 20 % of physicians were very familiar with these guidelines. Furthermore, 30 % had not heard of the guidelines, and 51 % had heard of them but were not familiar with their content.^40^ Interestingly, the study found that physicians who were unfamiliar with the guidelines were almost twice as likely to lack confidence in raising the issue of evaluating and recommending PA to their patients. The authors speculated that this lack of skill, knowledge, and self-efficacy in relation to guidelines might be attributed to the absence of any type of training on PA counseling among the evaluated physicians.

Therefore, reinforcing physicians’ ability to take a patient history, assess contraindications to PA practice, and prescribing PA may increase interest in the subject and the desire to acquire knowledge about PA. Encouraging regular PA training and practice can not only improve these skills but also benefit physicians’ health while enhancing the frequency and quality of PA counseling provided to patients. In this sense, facilitating health promotion and PA counseling by physicians can involve providing knowledge about current PA recommendations, encouraging regular PA practice, and offering learning tools to develop essential skills for PA counseling.

## Limitations

Findings should be interpreted with caution as the convenience sample does not entirely represent the reality of all hospitals in Brazil. The high complexity of the care provided by HCFMUSP results in limited time for preventive actions, which could have influenced the rates of willingness to assess and prescribe PA. Additionally, the low response rate could be attributed, at least in part, to the exhaustion of the institution’s medical staff during the COVID-19 pandemic. As this study used a convenience sample, it is possible that the physicians most interested in the subject were more likely to answer the questionnaire, potentially compromising the generalizability of the findings. Moreover, the self-reported nature of the data may have led to an overestimation of the participants’ level of PA and knowledge. Finally, it is important to acknowledge that data collection relied solely on the perspectives of the physicians, and a more comprehensive assessment that includes patients’ narratives or medical records may provide valuable, complementary data.

## Conclusion

**Fig. 3:**
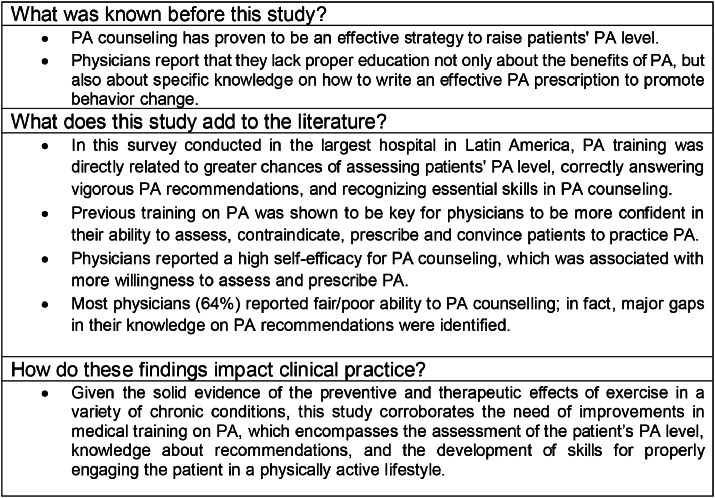
Previous evidence, main findings of this study and practical applications.

Although physicians recognize the importance of counseling PA and have high perceived self-efficacy in relation to their skills, a minority of them effectively assess and prescribe PA, and when they do, most do not provide accurate advice that aligns with current PA recommendations. This may be due to a lack of previous training on PA and/or lack of time for prevention measures, owing to the complexity of assistance inherent to a tertiary hospital. Given the solid evidence of the preventive and therapeutic effects of PA in a variety of chronic conditions, this study corroborates the need for improvements in medical training on PA, which includes the assessment of the patient’s PA level, knowledge about recommendations, and the development of skills for properly advising and engaging the patient on a physically active lifestyle (Fig. 3).

## CRediT authorship contribution statement

**Débora Borowiak Reiss:** Conceptualization, Methodology, Software, Investigation, Data curation, Writing – original draft, Visualization. **Ítalo Ribeiro Lemes:** Validation, Formal analysis, Data curation, Writing – review & editing. **Hamilton Roschel:** Writing – review & editing, Supervision. **Bruno Gualano:** Resources, Writing – review & editing, Supervision, Project administration.

## Declaration of competing interest

The authors declare no conflicts of interest.

## Appendix Supplementary materials

Image, application 1
