# Supplementary material for: Medical conduct and knowledge about physical activity counseling in the largest hospital complex in Latin America
Source: Clinics (Sao Paulo). 2025 May 7;80:100666. doi: 10.1016/j.clinsp.2025.100666 (PMC12133723; doi:10.1016/j.clinsp.2025.100666)
Supplement: Supplementary file 1 [file mmc1.docx]

**CLINICS-D-23-00613_ Supplementary Material**

Supplementary Material

| **Questionnaire on knowledge about physical activity recommendations** |
| --- |
|  |
| The aim of our work is to characterize clinical practice and medical knowledge about the recommendation of physical activity for chronically ill patients at Hospital das Clínicas da FMUSP, the largest hospital in Latin America. Below, you will find questions related to your education, your lifestyle habits and your specific knowledge about prescribing physical activity for chronically ill patients. |
|  |
| 1. Sex: ( ) F ( ) M |
|  |
| 2. Date of birth: __________ |
|  |
| 3. Date of answer: __________ (preenchimento automático REDCap) |
|  |
| 4. Age: _________________ (cálculo automático REDCap) |
|  |
| 5. Do you currently practice structured physical activity? |
| ( ) Yes ( ) No |
|  |
| 6. Has this changed with the onset of the COVID-19 pandemic? |
| ( ) Did not change. |
| ( ) Changed to more. |
| ( ) Changed to less. |
|  |
| 7 How many hours a day do you spend sitting (while awake)? |
| ( ) Up to 4 hours. |
| ( ) More than 4 to 8 hours. |
| ( ) More than 8 to 12 hours. |
| ( ) More than 12 to 16 hours. |
| ( ) More than 16 hours. |
|  |
| 8. Has this changed with the onset of the COVID-19 pandemic? |
| ( ) Did not change. |
| ( ) Changed to more. |
| ( ) Changed to less. |
|  |
| 9 In what year did you graduate in Medicine? __________ |
|  |
| 9.1 In which state did you graduate in Medicine? __________ |
|  |
| 10. Are you a resident? |
| ( ) Yes ( ) No |
|  |
| **If you answered “No”, go to question 11** |
|  |
| 10.1 Which specialty are you a resident of? __________ |
|  |
| 10.2 What year of residency are you in? |
| ( ) R1 ( ) R2 ( ) R3 ( ) R4 ( ) R5 |
| 11. Do you have specialization in any area? (If you present more than one, please respond in relation to the most recent training) |
| ( ) Yes ( ) No |
|  |
| 11.1 What? ________________ |
|  |
| 11.2 In what year did you complete your specialization? __________ |
|  |
| 12. Do you see patients: |
| ( ) Pediatric ( ) Adults and/or elderly ( ) Both |
|  |
| 13. During your training, did you have any type of trainning to recommend PA to your patients? |
| ( ) Yes ( ) No |
|  |
| 14. Do you exclusively work in any of these areas/specialties? |
| Yes ( ) No ( ) |
| (Anestesiology, Emergency Medicine, Forensic Medicine, Nuclear Medicine, Pathology, Laboratory Medicine, Radiology, Radiotherapy, Pediatric Emergency, Intensive Care, Pediatric Intensive Care, Neonatology, Digestive Endoscopy, Gynecological Endoscopy, Fetal Medicine, Palliative Care and Forensic Psychiatry). |
|  |
| **(If yes, it caracterizes exclusion criteria.)** |
|  |
| 15. Do you routinely assess your patients’ level of PA and sedentary time? |
| ( ) Always |
| ( ) Most of the time |
| ( ) Rarely |
| ( ) Never |
|  |
| 16. Has this changed with the onset of the COVID-19 pandemic? |
| ( ) Did not change. |
| ( ) Changed to more. |
| ( ) Changed to less. |
|  |
| 17. Do you think it is important to recommend PA for patients with chronic diseases? |
| ( ) Yes, the practice of PA can be a first choice treatment in some diseases. |
| ( ) Yes, the practice of PA can help as an adjuvant treatment. |
| ( ) No, the patients can even practice PA if They wish, but it does not interfere with the treatment. |
| ( ) No, the practice of PA can harm the treatment. |
|  |
| 18. Do you recommend PA for your patients with chronic diseases? |
| ( ) Always |
| ( ) Most of the time |
| ( ) Rarely |
| ( ) Never |
|  |
| 19. Has this changed with the onset of the Covid-19 pandemic? |
| ( ) Did not change. |
| ( ) Changed to more. |
| ( ) Changed to less. |
|  |
| **If you answered “Always” to question 18, skip to question 21.** |
|  |
| 20. List the reasons why you no longer recommend PA for patients with chronic diseases: |
| ( ) I don’t have enough time during my appointments. |
| ( ) I don’t have enough trainning for it. |
| ( ) I do not consider this to be part of my job. |
| ( ) I do not consider it a clinically importante parameter. |
| ( ) I believe that physical activity can worsen some symptoms of the disease. |
| ( ) Patients do not adhere to the recommendations. |
| ( ) Most of my patients have contraindications to the practice of PA. |
| ( ) I believe that patients have nowhere to do practice PA. |
| ( ) Others __________. |
|  |
| 21. Rate you ability in: |
| 21.1 Collect history of PA. |
| ( ) 1: Poor ( ) 2: Regular ( ) 3: Good ( ) 4: Excellent |
|  |
| 21.2 aAssess whether a patient has contraindications to exercise. |
| ( ) 1: Poor ( ) 2: Regular ( ) 3: Good ( ) 4: Excellent |
|  |
| 21.3 Write na exercise prescription. |
| ( ) 1: Poor ( ) 2: Regular ( ) 3: Good ( ) 4: Excellent |
|  |
| 21.4 Convincing a patient to engage in regular PA when appropriate. |
| ( ) 1: Poor ( ) 2: Regular ( ) 3: Good ( ) 4: Excellent |
|  |
| 22. Answer the questions below about PA recommendations for adults and elderly: |
| a) At a minimum, how many minutes of moderate PA should be practiced over the course of a week? __________ |
| a) Alternatively, to obtain the same benefits of moderate PA, how many minutes of vigorous PA should be practiced over a week? __________ |
| c) Strength exercises should be practiced at least how many days a week? __________ |
|  |
| 23. Answer the questions below about PA for children and adolescents: |
| a) At least how many minutes of moderate to vigorou PA should be practiced per day? __________ |
| b) For children aged 6 to 10, sedentary time during leisure time (TV, computer, cell phone, etc) should be limited to how many minutes per day? __________ |
| c) For adolescentes aged 11 to 18, sedentary time during leisure time (TV, computer, cell phone, etc) should be limited to how many minutes per day? __________ |
|  |
| 24. Check the correct alternatives: |
| ( ) PA should not be recommended for patients with chronic pain or funcional limitations. |
| ( ) Strength exercises should not be recommended for patients younger than 12 years old. |
| ( ) A person who does not reach the PA recommendations is considered sedentary. |
| ( ) PA should not be recommended for patients who have fever, leucopenia, anemia or malnutrition. |
| ( ) PA should be recommended for patients with chronic diseases, but sports praticies should not. |
| ( ) PA of very low intensity, such as light walking, promotes health benefits. |
| ( ) PA should not be recommended for patients with hypertension, type 2 diabetes and heart failure. |
| ( ) PA is any bodily movement produced by a muscle contraction that results in energey expenditure above baseline levels. |
| ( ) Patients with chronic disesases shoud always avoid sedentary behavior, except in periods of clinical descompensation |
| ( ) The exacerbation of inflammation and muscle damage are adverse effects of PA in patients with chronic diseases. |
|  |
| 25. Do you consider that trainning in evaluation and recommendation of PA should be part of the academic and professional training of general practitioners? |
| ( ) Must be a mandatory topic. |
| ( ) It can be an optional theme. |
| ( ) No. |
